# Two Randomized Controlled Trials of Bacillus Calmette-Guérin Vaccination to reduce absenteeism among health care workers and hospital admission by elderly persons during the COVID-19 pandemic: A structured summary of the study protocols for two randomised controlled trials

**DOI:** 10.1186/s13063-020-04389-w

**Published:** 2020-06-05

**Authors:** Thijs ten Doesschate, Simone J. C. F. M. Moorlag, Thomas W. van der Vaart, Esther Taks, Priya Debisarun, Jaap ten Oever, Chantal P. Bleeker-Rovers, Patricia Bruijning Verhagen, Arief Lalmohamed, Rob ter Heine, Reinout van Crevel, Janneke van de Wijgert, Axel B. Janssen, Marc J. Bonten, Cornelis H. van Werkhoven, Mihai G. Netea, on behalf of the BCG-CORONA study team#

**Affiliations:** 1 University Medical Center, Utrecht, The Netherlands; 2 Radboud University Medical Center, Nijmegen, the Netherlands

**Keywords:** COVID-19, Randomised controlled trial, protocol, BCG-vaccine, Health-Care Workers, Elderly

## Abstract

**Objectives:**

The objectives of these two separate trials are: (1) to reduce health care workers (HCWs) absenteeism; and (2) to reduce hospital admission among the elderly during the COVID-19 pandemic through BCG vaccination.

**Trial design:**

Two separate multi-centre placebo-controlled parallel group randomized trials

**Participants:**

(1) Health care personnel working in the hospital or ambulance service where they will take care of patients with the COVID-19 infection and (2) elderly ≥60 years. The HCW trial is being undertaken in 9 hospitals. The elderly trial is being undertaken in locations in the community in Nijmegen, Utrecht, and Veghel, in the Netherlands, using senior citizen organisations to facilitate recruitment.

**Intervention and comparator:**

For both trials the intervention group will be randomized to vaccination with 0.1 ml of the licensed BCG vaccine (Danish strain 1331, SSI, Denmark, equivalent to 0.075 mg attenuated *M. bovis*). The placebo group consists of 0.1 ml 0.9% NaCl, which is the same amount, and has the same colour and appearance as the suspended BCG vaccine.

**Main outcomes:**

(1) Number of days of unplanned work absenteeism in HCWs for any reason which can be continuously measured on a bi-weekly basis, and (2) the cumulative incidence of hospital admission due to documented COVID-19.

**Randomisation:**

Participants will be randomized to BCG vaccine or placebo (1;1) centrally using a computer- based system, stratified by study centre.

**Blinding (masking):**

Subjects, investigators, physicians and outcome assessors are blinded for the intervention. Only the pharmacist assistant that prepares- and research personnel that administers- study medicines are unblinded.

**Numbers to be randomised (sample size):**

(1) The sample size for the first trial is N=1500 HCWs randomised 1:1 to either BCG vaccine (n=750) and placebo (n=750) and (2) The sample size for the second trial is N=1600 elderly persons randomised to BCG vaccine (n=800) and the placebo group (n=800).

**Trial Status:**

HCW: version 4.0, 24-04-2020. Recruitment began 25-03-2020 and was completed on the 23-04-2020. Elderly: version 3.0, 04-04-2020. Recruitment began 16-04- 2020 and is ongoing.

**Trial registration:**

The HCWs trial was registered 31-03-2020 at clinicaltrials.gov (identifier: NCT04328441) and registered 20-03-2020 at the Dutch Trial Registry (trialregister.nl, identifier Trial NL8477). The elderly trial was registered 22-04-2020 at the Dutch trial registry with number NL8547.

**Full protocol:**

The full protocols will be attached as additional files, accessible from the Trials website (Additional file 1). In the interest in expediting dissemination of this material, the familiar formatting has been eliminated; this Letter serves as a summary of the key elements of the full protocol.

## Supplementary information

**Additional file 1.** Full study protocol.

## Data Availability

The investigators from the UMC Utrecht will have access to the final trial dataset of the HCWs trial and the investigators from the Radboudumc will have access to the final trial dataset of the Elderly trial. For the HCWs trial the full dataset will be made available with online electronic Case Report Form (eCRF) ResearchOnline, and for the Elderly trial with Castor.

