# Correction to: Two Randomized Controlled Trials of Bacillus Calmette-Guérin Vaccination to reduce absenteeism among health care workers and hospital admission by elderly persons during the COVID-19 pandemic: A structured summary of the study protocols for two randomised controlled trials

**DOI:** 10.1186/s13063-020-04521-w

**Published:** 2020-06-22

**Authors:** Thijs ten Doesschate, Simone J. C. F. M. Moorlag, Thomas W. van der Vaart, Esther Taks, Priya Debisarun, Jaap ten Oever, Chantal P. Bleeker-Rovers, Patricia Bruijning Verhagen, Arief Lalmohamed, Rob ter Heine, Reinout van Crevel, Janneke van de Wijgert, Axel B. Janssen, Marc J. Bonten, Cornelis H. van Werkhoven, Mihai G. Netea, on behalf of the BCG-CORONA study team#

**Affiliations:** 1 University Medical Center, Utrecht, The Netherlands; 2 Radboud University, Medical Center, Nijmegen, the Netherlands

**Correction to: Trials (2020) 21:481**

**https://doi.org/10.1186/s13063-020-04389-w**

Following publication of the original article [1], we have been notified that two names have been misspelled in the Acknowledgements section:

- Incorrect names:
  Claudia Recanititi
  Arief Lalmohammed
- Correct names:
  Claudia Recanatini
  Arief Lalmohamed
