# Supplementary material for: Two Randomized Controlled Trials of Bacillus Calmette-Guérin Vaccination to reduce absenteeism among health care workers and hospital admission by elderly persons during the COVID-19 pandemic: A structured summary of the study protocols for two randomised controlled trials
Source: Trials. 2020 Jun 5;21:481. doi: 10.1186/s13063-020-04389-w (PMC7273375; doi:10.1186/s13063-020-04389-w)
Supplement: Supplementary file 1 — Additional file 1. Full study protocol. [file 13063_2020_4389_MOESM1_ESM.docx]

**Two Randomized Controlled Trials of Bacillus Calmette-Guérin Vaccination to reduce absenteeism among health care workers and hospital admission among elderly during COVID-19 pandemic**

Thijs ten Doesschate*^1&^, Simone J. C. F. M. Moorlag*^2^, Thomas W. van der Vaart^1^, Esther Taks^2^, Priya Debisarun^2^, Jaap ten Oever^2^, Chantal P. Bleeker-Rovers^2^, Patricia Bruijning-Verhagen^1^, Arief Lalmohamed^1^, Rob ter Heine^2^, Reinout van Crevel^2^, Janneke van de Wijgert^1, 3^, Axel B. Janssen^1^, Marc J. Bonten^1^, Cornelis H. van Werkhoven**^1^ & Mihai G. Netea**^2, 4, 5^ - on behalf of the BCG-CORONA study team#

*shared first authorship

**shared senior authorship

&corresponding author, [t.tendoesschate@umcutrecht.nl](mailto:t.tendoesschate@umcutrecht.nl)

1. University Medical Center Utrecht, Utrecht University, Department of medical microbiology, The Julius Center for Health Sciences and Primary Care, Department of Clinical Pharmacy, Utrecht, The Netherlands

2. Radboud university medical center, Radboud Institute for Health Sciences, Department of Pharmacy, Department of Internal Medicine, Nijmegen, The Netherlands.

3. Institute of Infection and Global Health, University of Liverpool, Liverpool, United Kingdom

4. Craiova University of Medicine and Pharmacy, Human Genomics Laboratory, Craiova, Romania

5. University of Bonn, Department for Genomics & Immunoregulation, Life and Medical Sciences Institute (LIMES), Bonn, Germany

**BCG-CORONA study team:**

Canisius Wilhelmina Ziekenhuis, Nijmegen - Andreas Voss

Erasmus Medical Center, Rotterdam - Bart J. A. Rijnders, Stijn Klijn

Hagaziekenhuis, The Hague – Cees van Nieuwkoop

Jeroen Bosch Ziekenhuis, Den Bosch - Angèle Kerckhoffs

Leiden University Medical Center, Leiden – Anna H. Roukens, Vincent P. Kuiper, Jan Pieter R. Koopman

Noordwest Ziekenhuisgroep, Alkmaar - Wim J. A Boersma, Nienke Paternotte

Radboud University Medical Center, Nijmegen - Simone J. C. F. M. Moorlag, Esther Taks, Priya Debisarun, Rob ter Heine, Jaap ten Oever, Chantal P. Bleeker-Rovers, Reinout van Crevel, Mihai G. Netea

Sint Maartenskliniek, Nijmegen - Karin Veerman

University Medical Center Utrecht, Utrecht - Thijs ten Doesschate, Thomas W. van der Vaart, Claudia Recanatini, Patricia Bruijning-Verhagen, Arief Lalmohamed, Janneke van de Wijgert, Axel B. Janssen, Marc J. Bonten, Cornelis H. van Werkhoven

**Abstract:**

**Background:**

SARS-CoV-2 spreads rapidly throughout the world, mainly affecting elderly. The pandemic challenges the available hospital capacity, which is augmented by absenteeism of healthcare workers (HCWs). To safeguard continuity of patient care, strategies are needed to protect elderly and HCWs from (severe) COVID-19. Bacille Calmette-Guérin (BCG) is a vaccine against tuberculosis, with protective non-specific effects against other respiratory tract infections and viral infections. We hypothesize that BCG vaccination can reduce hospital admission among elderly and HCWs absenteeism during the COVID-19 pandemic.

**Methods and design:**

Two separate placebo-controlled randomized controlled trials are being conducted in the Netherlands. The first is a trial among HCWs in 9 hospitals and their affiliated ambulance services with as primary endpoint the reduction of absenteeism. The second is a trial among community-dwelling elderly (≥ 60 years) with cumulative incidence of COVID-19 related hospital-admission as primary endpoint. In both trials, participants are randomized to BCG vaccine and placebo (1:1). Subjects are being followed during a maximum of 180 days after randomization using a mobile application to register symptoms, health care visits, and work absenteeism (for HCWs). An adaptive design was chosen for both trials with frequent interim analyses of the primary endpoint. Trials were registered at the Dutch trial registry with numbers NL8477 (HCWs) and NL8547 (Elderly).

**Discussion:**

Randomized controlled trials are indispensable to evaluate the protective non-specific effects of BCG vaccine to prevent (severe) COVID-19. Based on previous experience and randomized controlled trials in adults and elderly, risks of BCG vaccine are considered low. The adaptive design enhances trial efficiency, allowing rapid informing of policy makers during the ongoing pandemic.

**Background:**

On 30 December 2019, a novel enveloped RNA beta-coronavirus was detected from a patient with pneumonia of unknown etiology in Wuhan, the capital city of Hubei province. The pathogen was named the severe acute respiratory syndrome coronavirus-2 (SARS-CoV-2), and the disease caused by it was termed coronavirus disease 2019 (COVID-19).^1,2^ COVID-19 spread rapidly throughout China and the rest of the world, with the first detected case in the Netherlands reported on 27 February 2020, a mere 8 weeks after the first reports of the disease.

The COVID-19 pandemic provides society with a formidable challenge. Especially elderly are at risk for a severe disease course and hospitalization.^3^ The number of required hospital admissions of SARS-CoV-2 infected patients is expected to outweigh health care capacities, as happened in the district of Wuhan, China. This will compromise quality of healthcare, facilitate nosocomial transmission and increase case fatality.^4^ Increased absenteeism of healthcare workers due to COVID-19 would lead to even greater deficits in qualified staff able to take care of patients.

Strategies to prevent SARS-CoV-2 infection or to mitigate its clinical consequences are, therefore, needed. To date, treatment for COVID-19 consists of supportive measures, with or without experimental therapies, although no significantly proven curative and preventive treatments have been identified.^5^

Bacillus Calmette-Guérin (BCG) was developed as a vaccine against tuberculosis, but studies have shown its ability to induce potent protection against other infectious diseases: the so called non-specific effects (NSEs).^6^ These non-specific beneficial effects of BCG vaccine are thought to be due to epigenetic and metabolic reprogramming of innate immune cells such as myeloid cells and NK cells, leading to an increased antimicrobial activity, a process termed ‘trained immunity’ or innate immune memory.^7^ Upon stimulation with a pathogen, the innate immune system is primed and able to react faster and more efficient to a secondary (related or non-related) stimulus. In experimental studies, BCG vaccine protects not only against bacterial and fungal infections, but also against viral infections such as influenza.^8^ Furthermore, among humans exposed to the live attenuated yellow fever vaccine virus, randomized to BCG vaccine one month earlier yielded less viremia and improved anti-viral responses as compared to placebo.^9^ The observed effects are proposed to a result of modulation of the human innate immune system through ‘trained immunity’ and are long-lasting for at least one year.^11,12^

In clinical studies, BCG vaccine was associated with less child mortality, mainly as a result of reduced neonatal sepsis, respiratory infections, and fever. ^10–12^ NSEs of BCG vaccine are not limited to children, although effects in adults are primarily based on *in vitro* data. In 2014, the WHO recommended performing high quality randomized controlled trials (RCT) to quantify the beneficial non-specific effects of BCG vaccine in humans.^13^ A meta-analysis of three RCTs revealed a 38% reduction in neonatal mortality (95%CI=0.17-0.54).^14^ Furthermore, an Indonesian trial performed in elderly has shown that BCG vaccine reduced the incidence of acute upper tract respiratory infections by 80% (95%CI=22-95%).^15^ BCG vaccination in immunocompetent adults is considered safe, even in the presence of latent infection or after prior BCG vaccination.^16,17^ In a RCT that compared revaccination with BCG versus placebo, no vaccine-related serious adverse events were observed in the 312 patients in the BCG vaccine arm. ^18^

**Objectives**

The trial among HCWs has the primary objective to reduce absenteeism among HCWs during COVID-19 pandemic. Secondary objectives of this trial are to reduce the cumulative incidence of documented COVID-19, hospital admission, ICU admission, or death, and to reduce the number of days of self-reported fever (≥38 degrees Celsius) or the number of days of self-reported fever (≥38 degrees Celsius) or acute respiratory symptoms.

The trial among elderly has the primary objective to reduce COVID-19-related hospital admission among elderly ≥60 years of age. Secondary objectives of this trial are to reduce the cumulative incidence of documented COVID-19, ICU admission, or death, and to reduce the number of days of self-reported fever (≥38 degrees Celsius) or self-reported acute respiratory symptoms.

If BCG vaccine appears to be efficacious, strategies for implementation in health care will be discussed with the competent authorities and institutions. In that scenario, BCG vaccine could serve as a rapidly available and widely applicable measure to protect the affected population during future epidemics.

**Methods/Design**

Both trials are multicenter investigator initiated pragmatic trials. The trial in HCWs is coordinated by the University Medical Center Utrecht (UMCU). Study subjects are recruited through nine hospitals in the Netherlands and their affiliating ambulance services. HCWs are invited to visit a study facility in the hospital for information and to provide informed consent.

The trial in elderly is coordinated by the Radboud University Medical Center Nijmegen (Radboudumc). Community-dwelling subjects are recruited through advertisements in local newspapers and local senior’s organizations. Candidate subjects can contact local study teams for additional information, and those meeting eligibility criteria are invited to visit a study location outside the hospital in Nijmegen and Utrecht.

*Population*

For the HCWs trial, participants will be recruited from 9 hospitals throughout the Netherlands, of which four academic hospitals and five middle to large teaching hospitals, see Figure 1. Hospitals were selected if they provided care for CoVID-19 patients, and if they had the willingness and capacity to set up an investigation in a short time. For the elderly trial, participants will be recruited at three central locations outside the hospital in the Netherlands, see Figure 1. Community-dwelling elderly can visit one of these sites by car through a drive-in or by visiting the outpatient clinic. In order to get a representative population, special attention has been generated to the recruitment of elderly people aged 80 or older or with a migration background.

Eligibility criteria for both trials can be found in Table 1. These criteria are designed to guarantee the participant’s safety. To prevent absenteeism due to extensive local reactions in HCWs with prior BCG vaccination, subjects that experienced such reactions previously were excluded.

Figure 1: Locations of recruitment for the HCWs trial and the elderly trial


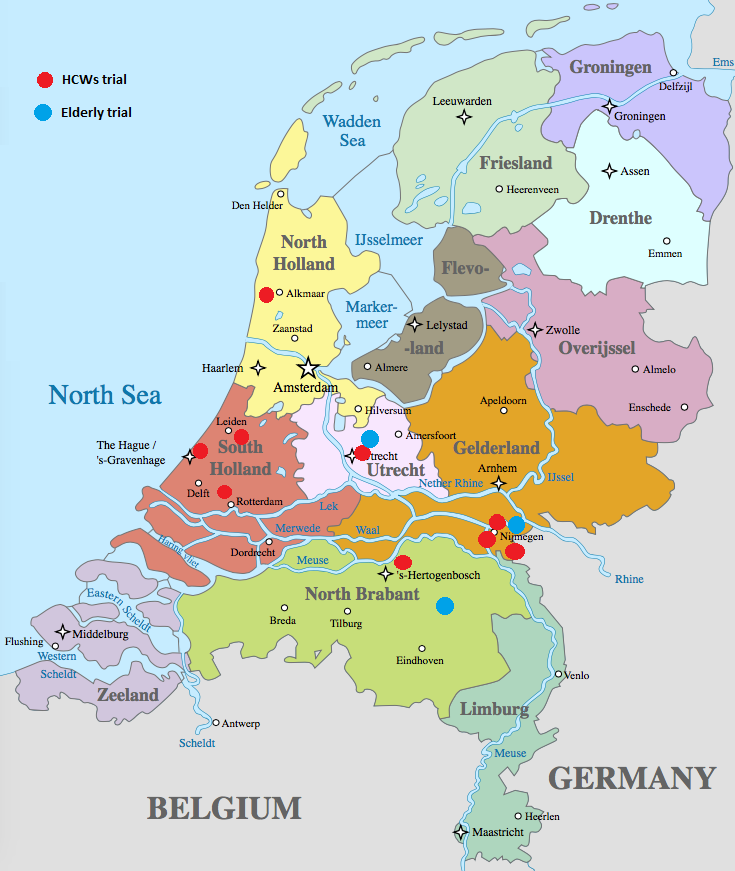


**Table 1:** Eligibility criteria for the BCG vaccine trials involving Health Care Workers and elderly.

| Eligibility criteria: | Applies to |
| --- | --- |
| *Inclusion criteria* | |
| ≥60 years | *Elderly trial* |
| ≥18 years of age | *HCWs trial* |
| Health care workers (expected to) take care for patients with COVID-19 infection | *HCWs trial* |
| In possession of a smartphone | *HCWs trial* |
| *Exclusion criteria* | |
| Fever (>38 ºC) within the past 24 hours | *Both trials* |
| Suspicion of current active viral or bacterial infection | *Both trials* |
| Severely immunocompromised subjects* | *Both trials* |
| Active solid or non-solid malignancy or lymphoma within the prior two years | *Both trials* |
| Known allergy to (components of) the BCG vaccine or serious adverse events to prior BCG vaccine administration | *Both trials* |
| Known active or latent Mycobacterium tuberculosis or with another mycobacterial species. A history with- or a suspicion of M. tuberculosis infection.** | *HCWs trial* |
| Pregnancy | *HCWs trial* |
| Direct involvement in the design or the execution of the BCG-CORONA study | *HCWs trial* |
| Expected absence from work of ≥4 of the following 12 weeks due to any reason (holidays, maternity leave, retirement, planned surgery etc) | *HCWs trial* |
| Employed to the hospital < 22 hours per week | *HCWs trial* |
| Vaccination in the past 4 weeks or expected vaccination during the study period, independent of the type of vaccination. | *HCWs trial* |
| Expected vaccination during the first three months of the study period | *Elderly trial* |
| Active participation in another research study that involves BCG vaccine administration | *Elderly trial* |

HCWs= Health care workers, BCG= Bacille Calmette-Guerin, COVID-19= Coronavirus Disease-2019

* This exclusion category comprises: a) subjects with known infection by the human immunodeficiency virus (HIV-1); b) neutropenic subjects with less than 500 neutrophils/mm3; c) subjects with solid organ transplantation; d) subjects with bone marrow transplantation; e) subjects under chemotherapy; f) subjects with primary immunodeficiency; g) severe lymphopenia with less than 400 lymphocytes/mm3; h) treatment with any immunosuppressant drugs such as anti-cytokine therapies, and treatment with oral or intravenous steroids defined as daily doses of 10mg prednisone or equivalent for longer than 3 months, or probable use of oral or intravenous steroids in the following four weeks

** A latent Tuberculosis infection is defined as having a positive Mantoux or Quantiferon test with known risk factors for Tuberculosis, that is not yet been treated with antimicrobials.

*Sample size*

For HCWs, a decrease in number of days of unplanned absenteeism for any reason of 20% was deemed relevant. With an expected absenteeism in the control group of 5%, a sample size of 1000 subjects will be required with follow-up until conclusion of less than 12 weeks. The 5% absenteeism is based on the average hospital absenteeism during the winter season. Computer simulations were used to determine the required sample size and expected follow-up duration. Due to extensive willingness among HCWs to participate in the study, the desire to provide a conclusive result as early as possible, and the assurance of sufficient BCG vaccine, it was decided to enroll 750 subjects per intervention group.

The trial among elderly was set-up as an endpoint-driven trial, i.e. the follow-up of the study will continue until the predefined number of endpoints is reached. The course of the COVID-19 pandemic is unknown and may be influenced by national policy changes. The advantage of an endpoint-driven trial is that the power of the trial is minimally affected, even if the incidence of the primary outcome is lower than assumed; the follow-up duration will in that case be prolonged to reach the number of endpoints. We assumed that a total of 50% of the control group will get COVID-19 and of these, 15% will be hospitalized. If the cumulative incidence of the primary endpoint is assumed to be 7.5%, a 50% relative reduction of the primary endpoint will be reached in the BCG vaccine group. We use a two-sided alpha of 0.05 and aim for 90% power. With sequential interim analyses, we require a maximum total of 103 primary endpoints. We aim to enroll 800 subjects per intervention group.

*Study design*

HCWs will be randomized to receive BCG vaccine or placebo in a 1:1 ratio. Randomization will be performed using a computer generated dynamic randomization algorithm. Subjects, investigators, physicians and outcome assessors are blinded for the intervention. Only the pharmacist assistant that prepares- and research personnel that administers- study medicines are unblinded. Baseline study data will be entered in the online electronic Case Report Form (eCRF) ResearchOnline by the investigating personnel. ^19^

Elderly will also be randomized to receive BCG vaccine or no vaccine in a 1:1 ratio. Randomization will be performed using a computer generated dynamic randomization algorithm. Subjects and outcome assessors will be blinded for the intervention. The pharmacist assistant that prepares- and research personnel that administers- study medicines are unblinded. Baseline study data will be entered in the online electronic Case Report Form (eCRF) Castor by the investigating personnel. ^20^

In both studies subject follow-up will be done using a mobile application, the ResearchFollowApp.^21^ On a daily basis, subjects will be asked to fill in a questionnaire regarding COVID-19 symptoms, adverse events and – only for HCWs – absenteeism, and on a weekly basis subjects will be asked to fill in a questionnaire regarding COVID-19 exposure and health care visits. If the application is not filled in for seven days, subjects will receive a push notification, and the investigator receives a notification by email. If a subject registers hospital admission in the application, the investigator receives a notification by email. Elderly without smartphone will be approached by telephone weekly with follow-up questions. The duration of follow-up in both trials depends on the results of the interim analysis with a maximum of 180 days.

Blood samples will be collected from HCWs in three participating centers, at 12 weeks after enrollment and at the end of the study, and from elderly 6 months after enrollment. Samples will be used to determine COVID-19 serology status, and for characterization of immunological phenotypes at several levels. Immune cell subpopulations will be assessed by flow cytometry, while cytokine production capacity in peripheral blood mononuclear cells will be measured after stimulation with bacterial and viral stimuli. The trained immunity phenotype will also be assessed at transcriptional and epigenetic level by RNA and Assay for Transposase-Accessible Chromatin with high throughput sequencing (ATAC) sequencing of monocyte populations.

Infection prevention measures are being taken in the logistics of the trial. In the HCWs trial these measures meet the hospital's infection prevention requirements. In the elderly trial, study personnel wears gloves and a mouth mask.

*Treatment arms*

Subjects in both trials that are allocated to the intervention group will be vaccinated with 0.1 ml of the licensed BCG vaccine (Danish strain 1331, SSI, Denmark, equivalent to 0.075 mg attenuated *M. bovis*) using the standard vaccination technique for this vaccine with intradermal injection in the left upper arm. Subjects in both trials that are allocated to placebo will receive a placebo consisting of 0.1 ml 0.9% NaCl, which is the same amount, and has the same color as the suspended BCG vaccine. Persons are non-eligible if they use immunosuppressive medicines or if they expect to require a vaccine within the study period (see eligibility criteria). There are no known other interactions between BCG vaccine and other drugs. Subjects can leave the study at any time for any reason if they wish to do so without any consequences. A participant will only be replaced in case of withdrawal before the administration of BCG vaccine/placebo.

*Endpoints and baseline characteristics*

The primary and secondary endpoints of the two trials are listed in Table 2. The secondary and exploratory endpoints will be assessed at the end of the study, which is adaptive on the interim results of the primary endpoint, see the paragraph regarding statistical analysis. The baseline characteristics to be collected and follow-up questionnaires are listed in supplementary material A and B.

One of the secondary endpoints in the HCW trial is documented COVID-19 infection. It is important to keep in mind that health workers in the Netherlands are structurally examined for the presence of COVID-19 in case of any respiratory symptoms or in case of fever. Therefore, we expect a high detection rate.

**Table 2:** Primary and secondary endpoints for the HCWs and the elderly trial.

| Primary endpoint HCWs trial | Primary endpoint Elderly trial |
| --- | --- |
| Number of days of unplanned work absenteeism for any reason | The cumulative incidence of hospital admission due to documented COVID-19 |
| Secondary endpoints used in both trials | |
| the cumulative incidence of documented COVID-19 | |
| the cumulative incidence of self-reported acute respiratory symptoms or fever | |
| the cumulative incidence of death due to documented COVID-19 | |
| the cumulative incidence of Intensive Care Admission due to documented COVID-19 | |
| Secondary endpoints HCWs trial | **Secondary endpoints Elderly trial** |
| the cumulative incidence of Hospital Admission due to documented COVID-19 | the cumulative incidence of Hospital Admission for any reason |
| the number of days of unplanned absenteeism, because of documented COVID-19 | the duration of hospital admission due to documented COVID-19 |
| Exploratory endpoints used in both trials | |
| the number of days of self-reported fever (≥38 gr C) | |
| the cumulative incidence of self-reported fever (≥38 gr C) | |
| the number of days of self-reported acute respiratory symptoms | |
| the cumulative incidence of self-reported acute respiratory symptoms | |
| the cumulative incidence of death for any reason | |
| the cumulative incidence of Intensive Care Admission for any reason | |
| the incidence and magnitude of plasma/serum antibodies (IgA,M,G) and COVID-19-specific antibodies at the end of the study period | |
| Exploratory endpoints HCWs trial | **Exploratory endpoints Elderly trial** |
| the number of days of unplanned absenteeism because of self-reported acute respiratory symptoms | the cumulative incidence of delirium due to COVID-19 |
| the number of days of absenteeism, because of imposed quarantine as a result of exposure to COVID-19 | the cumulative incidence of pneumonia, skin infection, urinary tract infection, flu and gastro-intestinal infection |
| the number of days of absenteeism, because of imposed quarantine as a result of having acute respiratory symptoms, fever or documented COVID-19 | the duration of hospital admission for any reason |
| the cumulative incidence of hospital admission for any reason |  |

**Statistical analysis:**

*Data and Safety Monitoring Board (DSMB)*For both trials, the independent DSMB consists of three experts, i.e. a statistician, an infectious disease specialist and a medical microbiologist. The DSMB statistician is involved in the initial review of the data, together with the trial statistician. Data analysis will be replicated independently by the DSMB statistician, in case of superiority or futility.

*HCWs trial*

For the HCWs trial, the primary endpoint, work absenteeism for any reason, will be reported as the average proportion of sick-days with standard deviation by treatment arm. It will be analysed as total counts (i.e. one observation per participant) using a Bayesian negative binomial regression with a fixed effect for BCG, hospital, and enrolment week (categorical), age, department (whether planned to work on COVID-19 dedicated department), sick leave prior to enrolment (as a proportion of fte), and presence of at least one of four comorbidities: cardiovascular disease, use of anti-diabetic medication, asthma, pulmonary diseases. Age and prior sick leave will be modelled using a spline function. The total number of planned workdays for the respective person over the follow-up period will be used as offset (log-transformed). The brm function from the R package brms will be used for fitting the negative binomial model.^21^

The effect will be reported as a relative risk with 95% and 99% CI. The posterior probability for the superiority hypothesis (RR < 1) will be reported as well as the posterior probability for the futility hypothesis (RR > 0.8).

Model assumptions will be checked using residuals plots and rootograms and posterior distribution plots will be presented. If model assumptions are violated, the following alternative model will be used: analysis as sick/non-sick working days (i.e. multiple observation per participant, one observation for each planned working day) using a Bayesian logistic regression model with a random intercept per participant, a fixed effect for BCG vaccine, hospital, and observation week (categorical), the same additional covariates as planned for the negative binomial model, and an autocorrelation structure of 1^st^ order over time within participants. This model yields an odds ratio (OR) which can be interpreted as RR if the risk of sick leave is <10% and will be used for determination of superiority or futility in the same manner as planned for the RR. The same modelling approach will be used during interim analyses, which will be performed starting from 4 weeks after the first enrolled patient and subsequently every 2 weeks. The interim analysis will be performed by the trial statistician unblinded and will not be disclosed to other study team members. Monthly, the results will be provided to the DSMB unblinded.

*Elderly trial*

For the primary endpoint hospital admission due to COVID-19 a competing events analysis (Fine & Gray model) will be used. ^22^ This will use time to admission as the dependent outcome, the study arm as the independent variable, with mortality as potential competing event, with a fixed effect for BCG vaccine, enrolment week (categorical), age, and the following comorbidities: cardiovascular disease, Diabetes Mellitus, pulmonary diseases, hypertension. Subjects will be censored at the end of their follow-up. We will use a symmetrical group sequential design with frequent interim analyses after reaching 25%, 50%, 75%, and 100% of the required number of events (= 26, 52, 78 and 103 events) using Pocock bounds. During interim analyses study groups will be denoted A and B (randomly linked to BCG vaccine or no treatment). The results of this interim analysis will be reported to the DSMB. Upon request of the DSMB the allocation can be revealed. The DSMB will use as guideline to recommend stopping the trial when the z-score of treatment group in the Fine & Gray model crosses on either side the boundary of 2.36, see supplementary material C for a graphical display.

*Secondary and exploratory endpoints of both trials*

Time to event data (such as the cumulative incidence of documented COVID-19 infection) will be analysed using a proportional hazards model or a Fine & Gray model, depending on the presence of competing events, count data (such as the number of days of self-reported fever) will be analysed using a negative binomial regression model. The analyses will be described in a statistical analysis plan.

**Ethical considerations**

The HCWs trial was approved by the medical ethics committee of the UMCU and the elderly trial by the medical ethics committee of the Radboudumc. Local approval was given by all participating centers. For both studies, the ethics committees assessed that the study possesses a negligible risk for subjects. Any harm caused by negligence will be covered by the liability insurance of each participating center. This covers additional health care and compensation.

In both trials, subjects are blinded. If needed, un-blinding is possible 24/7 by telephone. After completion of the trials, if superiority is demonstrated, subjects allocated to the control groups that have not experienced COVID-19 will be offered BCG vaccine if the endemic is still ongoing. The first 3 days of enrollment, subjects for the Elderly trial were unblinded pending approval for the use of placebo by the medical ethics committee of the Radboudumc.

Future amendments to the protocol will be reviewed by the ethics committee that approved the trial.

**Oversight and monitoring**

As sponsors, the UMCU and Radboudumc are responsible for the design, preparation, and conduct of the HCWs trial and elderly trial, respectively. Both studies will be monitored by monitors appointed by the sponsor with no involvement in the design or the execution of the trials.

For both trials, the first monitor visit will take place before inclusion of the first study participant. During this visit, the presence and completeness of the relevant Study Files will be checked. Additionally, the quality and competence will be evaluated of the personnel that will perform informed consent and who will administer the study medication. After inclusion of 10 participants at each participating site, the site will be monitored with a check of the completeness of 3 randomly chosen informed consent forms and the accuracy of the eligibility criteria. In case of findings, more intensive monitoring will take place. Thereafter, monitoring will be performed centrally by checking the completeness of follow-up data from participants. At the close-out visit, the presence and completeness of the relevant Study Files will be checked.

**Registration of the trials**

The HCWs trial was registered at clinicaltrials.gov (identifier: NCT04328441) and the Dutch Trial Registry (trialregister.nl, identifier Trial NL8477). Recruitment started on 25^th^ March 2020 and finished at 23 April 2020. The elderly trial was registered at the Dutch trial registry with number NL8547. Recruitment started at 16 April 2020 and is ongoing.

**Discussion**

These two investigator-initiated randomized controlled trials investigate the efficacy of BCG vaccine to protect people from suffering COVID-19 or severe symptoms resulting from this. The aim is to ensure the safety, health and fitness of HCWs and the elderly population.

Based on the capacity of BCG vaccine to reduce the incidence of respiratory tract infections in children, to exert antiviral effects in experimental models, and to reduce viremia in an experimental human model of viral infection, we hypothesize that BCG vaccination may induce (partial) protection against COVID-19 infection and/or the severe consequences of infection.

As primary endpoint for the HCWs study, we decided to use all-cause HCWs absenteeism. We selected secondary endpoints that have a more explanatory nature, e.g. the cumulative incidence of documented COVID-19. The aim of our trial is to ensure the continuity of health care, and this is the reason for this pragmatic primary endpoint. Moreover, BCG vaccine is expected to reduce all-cause infections and not only COVID-19. Moreover, this was a non-funded study, precluding intensive personal follow-up, and the measurement of absenteeism as this was considered relevant, and immediately obvious.

Participants of both trials were blinded for the study treatment they received, although a substantial percentage of persons is expected to develop a local reaction at the application site, which could lead to deblinding. Unfortunately, better blinding is impossible. For both trials, we selected objectively measurable primary endpoints that are not affected by deblinding.

Since the beginning of our trials, they have received overwhelming media attention and scientific interest from all over the world. As a consequence, three ecological studies and eleven RCT’s have been developed with more or less the same objectives, of which two in elderly and nine in HCWs. Moreover, we received questions about whether we recommend BCG vaccination for COVID-19 prophylaxis at this moment. As an answer, we would like to refer to the recent statement of the WHO in which it is not recommended to use BCG vaccine for the prevention of COVID-19 until more evidence exists for the efficacy of it, for example through our trials. ^24^ It is crucial to maintain BCG for regular indications, such as to protect neonates from tuberculosis.

Before we send out a press release, the results of our trial will be reported to the competent authorities and institutions in order to facilitate them to provide a response i.e. the board of directors of the affiliated hospitals, the fabricant of BCG, the National Institute for Public Health and Environment (RIVM), the European Medicine Agency (EMA) and the World Health Organization (WHO). Strategies for implementation of BCG vaccine in health care will be discussed with above parties, in case of efficacy and an ongoing epidemic.

**Authors' contributions**

The whole study team was involved by the design and conduction of the trial. The first version of the protocol manuscript was written by Thijs ten Doesschate and corrections to this draft were made by all study team members.

**Conflict of interest statements**

Authors declare to have no conflicts of interests.

**Role of funding source**

The funding for these investigator-initiated studies for the BCG injections and the design of the eCRF and the mobile application came from the UMCU and Radboudumc. The participating centers contributed without financial compensation. The authors received no external funding for the conduction of this paper.

**Acknowledgements**
We gratefully acknowledge all research personnel for their ongoing help in the conduction of these trials, e.g. research nurses, pharmacy assistants, data management, monitors, and the planning and secretarial support office.

**Supplementary material A**:

Baseline data collected for the HCWs trial

| **VARIABLE** | **DERIVATION** | **STATISTICAL METHOD *** |
| --- | --- | --- |
| Age (years) | Self-reported; baseline eCRF | Median (IQR) or mean (sd) ^1^ |
| Gender | Self-reported; baseline eCRF: Male/ Female | n/N (%) |
| Weight | Self-reported; baseline eCRF | kg |
| Length | Self-reported; baseline eCRF | kg |
| History of BCG vaccination | Self-reported; baseline eCRF: Yes/No | n/N (%) |
| Age at BCG vaccination | Self-reported; baseline eCRF | Median (IQR) or mean (sd) ^1^ among those with reported history of BCG vaccination |
| History of positive Mantoux test | Self-reported; baseline eCRF. Options are:  - Yes  - No; at least one negative test  - No; never tested  Answer option “I don’t know” will be handled as missing value | n/N (%) for all categories |
| History of positive TB quantiFERON test | Self-reported; baseline eCRF. Options are:  - Yes  - No; at least one negative test  - No; never tested  Answer option “I don’t know” will be handled as missing value | n/N (%) for all categories |
| Receipt of influenza vaccination in this winter season | Self-reported; baseline eCRF: Yes/No | n/N (%) |
| Receipt of other vaccinations in past 12 months | Self-reported; baseline eCRF (open question) ^2^ | n/N (%) for each reported vaccination |
| History of respiratory tract infection in past winter | Self-reported; baseline eCRF. Options are:  - Yes, with fever  - Yes, without fever  - No | n/N (%) for all categories: |
| Use of anti-hypertensive medication | Self-reported; baseline eCRF: Yes/No | n/N (%) |
| History of cardiovascular disease | Self-reported; baseline eCRF: Yes/No | n/N (%) |
| Use of anti-diabetic medication | Self-reported; baseline eCRF: Yes/No | n/N (%) |
| History of asthma | Self-reported; baseline eCRF: Yes/No | n/N (%) |
| History of other pulmonary diseases | Self-reported; baseline eCRF (open question) ^2^ | n/N (%) for each reported pulmonary disease |
| History of smoking | Self-reported; baseline eCRF. Options are:  - Never smoked  - Ever smoked  - Current smoking | n/N (%) for all categories |
| History of allergic rhinitis | Self-reported; baseline eCRF: Yes/No | n/N (%) |
| Number of household members | Self-reported; baseline eCRF | Median (IQR) or mean (sd) ^1^ |
| Age of household members | Self-reported; baseline eCRF | Mean (SD)  % with household members < 18  % with household members > 50 |
| Department of employment | Self-reported; baseline eCRF. Options are:  - Intensive care  - Medium care  - Emergency room  - Internal medicine  - Infectious diseases  - Pulmonary diseases  - Other, specify (open question)^2^ | n/N (%) for all categories |
| Position at department of employment | Self-reported; baseline eCRF. Options are:  - Nurse  - Medical doctor  - Paramedical  - Supportive  - Secretary | n/N (%) for all categories |
| Current or planned deployment in dedicated COVID-19 department | Self-reported; baseline eCRF. Yes/No  Answer option “I don’t know” will be handled as missing value | n/N (%) |
| Average number of working days in the hospital | Self-reported; baseline eCRF. | Median (IQR) or mean (sd) ^1^ |
| Average number of working days on evening/night shift | Self-reported; baseline eCRF. | Median (IQR) or mean (sd) ^1^ |
| Percentage of working time spend with direct patient contact | Self-reported; baseline eCRF. Options are:  - ≤25%  - 26-50%  - 51-75%  - >75% | n/N (%) for all categories |
| Has at least one day of work loss due to illness between 1 January and 15 March 2020 | Self-reported; baseline eCRF. Options are:  - Yes, due to respiratory tract infection  - Yes, for alternative health issue  - No | n/N (%) for all categories |
| Total days of work loss due to illness between 1 January and 15 March 2020 | Self-reported; baseline eCRF. | Median (IQR) or mean (sd) ^1^ among those with reported work loss due to illness |
| Previously tested for COVID-19 | Self-reported; baseline eCRF. Options are:  - No  - Yes, negative test result  - Yes, positive test result | n/N (%) for all categories |
| Number of days since negative test | Self-reported; baseline eCRF. Date negative test minus date of enrolment. | Median (IQR) or mean (sd) ^1^ among those with reported negative test result |
| Number of days since positive test | Self-reported; baseline eCRF. Date positive test minus date of enrolment. | Median (IQR) or mean (sd) ^1^ among those with reported positive test result |
| Has had negative COVID-19 test after being tested positive | Self-reported; baseline eCRF. Yes / No | n/N (%) |
| Number of days since negative test after being tested positive | Self-reported; baseline eCRF. Date negative test minus date of enrolment. | Median (IQR) or mean (sd) ^1^ among those with reported negative test result after being tested positive |

Daily questionnaire regarding symptoms for the HCWs trial

| Do you have any of the following complaints? | | Answers | | | | | |
| --- | --- | --- | --- | --- | --- | --- | --- |
| 1 | Fever | No | Hardly | A little | Moderate | A lot | Extremely much |
| 2 | Cough | No | Hardly | A little | Moderate | A lot | Extremely much |
| 3 | Sore throat | No | Hardly | A little | Moderate | A lot | Extremely much |
| 4 | Nose colds | No | Hardly | A little | Moderate | A lot | Extremely much |
| 5 | Short of breath | No | Hardly | A little | Moderate | A lot | Extremely much |
| 6 | Headache | No | Hardly | A little | Moderate | A lot | Extremely much |
| 7 | Muscle strain | No | Hardly | A little | Moderate | A lot | Extremely much |
| 8 | Cold shivers | No | Hardly | A little | Moderate | A lot | Extremely much |
| 9 | Fatigue | No | Hardly | A little | Moderate | A lot | Extremely much |
| 10 | Diarrhea | No | Hardly | A little | Moderate | A lot | Extremely much |

Daily questionnaire regarding absenteeism for the HCWs trial

| Question | | Answers | | |
| --- | --- | --- | --- | --- |
| 1 | Have you worked in the past day (regardless of day, evening, night)? | Yes | No, today was not a working day for me | No, I am absent from my planned work (continue question 2) |
| 2 | Why are you absent from your work? | I am too ill to work | I am not ill, but in quarantine because of minor complaints or COVID-19 exposure | |

Weekly questionnaire regarding health care visits and COVID-19 exposure*

| Question | | Answers | | | | |
| --- | --- | --- | --- | --- | --- | --- |
| 1 | Have you had an investigation on a COVID-19 infection in the past week?** | No | Yes, final result will follow | | Yes, COVID-19 demonstrated | Yes, COVID-19 not demonstrated |
| 2 | Have you consulted a doctor because of your own health in the past week?*** | No | | Yes | | |
| 3 | Have you been exposed to patients with COVID-19 in the past week? **** | No | | Yes | | |

*After 7 days participants are asked for the severity of local reaction at the application site
** If yes, what was the date of this investigation?
*** If yes, was this because of a respiratory tract infection? Were you hospitalized, and if so, on what date, and was this on the Intensive Care Unit and have you been discharged?
**** If yes, on what date(s) were you exposed to COVID-19?

**Supplementary material B**: baseline data collected for the elderly trial

| **VARIABLE** | **DERIVATION** | **STATISTICAL METHOD *** |
| --- | --- | --- |
| Age (years) | Self-reported; baseline eCRF | Median (IQR) or mean (sd) ^1^ |
| Gender | Self-reported; baseline eCRF: Male/ Female | n/N (%) |
| Weight | Self-reported; baseline eCRF | kg |
| Length | Self-reported; baseline eCRF | kg |
| Match between real age and estimated age | Reported by researcher; baseline eCRF | n/N (%) |
| Profession | Self-reported; baseline eCRF (open question) | n/N (%) |
| Receiving nursing aid | Self-reported; baseline eCRF. Options are:  - No  - Yes, for cleaning  - Yes, for groceries  - Yes, for activities outside the house  - Yes, for taking a shower  - Yes, for all personal care  - Yes, else | n/N (%) for all categories |
| History of BCG vaccination | Self-reported; baseline eCRF: Yes/No | n/N (%) |
| Age at BCG vaccination | Self-reported; baseline eCRF | Median (IQR) or mean (sd) ^1^ among those with reported history of BCG vaccination |
| History of positive Mantoux test | Self-reported; baseline eCRF. Options are:  - Yes  - No; at least one negative test  - No; never tested  Answer option “I don’t know” will be handled as missing value | n/N (%) for all categories |
| Follow up after positive Mantoux | Self-reported; baseline eCRF. Options are:  - Yes, X-thorax negative  -Yes, X-thorax positive  - Yes, QuantiFERON test negative  - Yes, QuantiFERON test positive  - No  Answer option “I don’t know” will be handled as missing value | n/N (%) for all categories |
| Receipt of influenza vaccination in previous winter season | Self-reported; baseline eCRF: Yes/No | n/N (%) |
| Receipt of other vaccinations in past 12 months | Self-reported; baseline eCRF (open question) ^2^ | n/N (%) for each reported vaccination |
| History of respiratory tract infection in past winter | Self-reported; baseline eCRF. Options are:  - Yes, with fever  - Yes, without fever  - No | n/N (%) for all categories: |
| History of hypertension | Self-reported; baseline eCRF: Yes/No | n/N (%) |
| History of cardiovascular disease | Self-reported; baseline eCRF: Yes/No | n/N (%) |
| History of Diabetes Mellitus | Self-reported; baseline eCRF: Yes/No | n/N (%) |
| History of asthma | Self-reported; baseline eCRF: Yes/No | n/N (%) |
| History of renal disease | Self-reported; baseline eCRF: Yes/No | n/N (%) |
| History of other pulmonary diseases | Self-reported; baseline eCRF (open question) ^2^ | n/N (%) for each reported pulmonary disease |
| History of other chronic diseases | Self-reported; baseline eCRF (open question) | n/N (%) for each reported disease |
| History of smoking | Self-reported; baseline eCRF. Options are:  - Never smoked  - Ever smoked  - Current smoking  -Smoker in household | n/N (%) for all categories |
| History of allergic rhinitis | Self-reported; baseline eCRF: Yes/No | n/N (%) |
| Known allergies | Self-reported; baseline eCRF (open question) |  |
| Use of medication | Self-reported; baseline eCRF: Yes/No | n/N (%) |
| In case of medication use: what kind of medication and in which dosage | Self-reported; baseline eCRF  (open question) | n/N (%) for all medication |
| Number of household members | Self-reported; baseline eCRF | Median (IQR) or mean (sd) ^1^ |
| Age of household members | Self-reported; baseline eCRF | Mean (SD)  % with household members < 18  % with household members > 50 |
| Illness between 27 February and 10 April 2020 | Self-reported; baseline eCRF. Options are:  - Yes, due to respiratory tract infection  - Yes, for alternative health issue  - No | n/N (%) for all categories |
| Total days of illness between 27 February and 10 April 2020 | Self-reported; baseline eCRF. | Median (IQR) or mean (sd) ^1^ |
| Contact with person that was tested positively for coronavirus | Self-reported; baseline eCRF.  Yes/No | n/N (%) |
| Previously tested for COVID-19 | Self-reported; baseline eCRF. Options are:  - No  - Yes, negative test result  - Yes, positive test result | n/N (%) for all categories |
| Number of days since negative test | Self-reported; baseline eCRF. Date negative test minus date of enrolment. | Median (IQR) or mean (sd) ^1^ among those with reported negative test result |
| Number of days since positive test | Self-reported; baseline eCRF. Date positive test minus date of enrolment. | Median (IQR) or mean (sd) ^1^ among those with reported positive test result |

Daily questionnaire regarding symptoms for the elderly trial

| Do you have any of the following complaints? | | Answers | | | | | |
| --- | --- | --- | --- | --- | --- | --- | --- |
| 1 | Fever | No | Hardly | A little | Moderate | A lot | Extremely much |
| 2 | Cough | No | Hardly | A little | Moderate | A lot | Extremely much |
| 3 | Sore throat | No | Hardly | A little | Moderate | A lot | Extremely much |
| 4 | Nose colds | No | Hardly | A little | Moderate | A lot | Extremely much |
| 5 | Short of breath | No | Hardly | A little | Moderate | A lot | Extremely much |
| 6 | Headache | No | Hardly | A little | Moderate | A lot | Extremely much |
| 7 | Muscle strain | No | Hardly | A little | Moderate | A lot | Extremely much |
| 8 | Cold shivers | No | Hardly | A little | Moderate | A lot | Extremely much |
| 9 | Fatigue | No | Hardly | A little | Moderate | A lot | Extremely much |
| 10 | Diarrhea | No | Hardly | A little | Moderate | A lot | Extremely much |

Weekly questionnaire regarding health care visits and COVID-19 exposure*

| Question | | Answers | | | | |
| --- | --- | --- | --- | --- | --- | --- |
| 1 | Have you had an investigation on a COVID-19 infection in the past week?** | No | Yes, final result will follow | | Yes, COVID-19 demonstrated | Yes, COVID-19 not demonstrated |
| 2 | Have you consulted a doctor because of your own health in the past week?*** | No | | Yes | | |
| 3 | Have you been exposed to patients with COVID-19 in the past week? **** | No | | Yes | | |

*After 7 days participants are asked for the severity of local reaction at the application site
** If yes, what was the date of this investigation?
*** If yes, did the doctor found an infection and what kind of infection? Were you hospitalized, and if so, on what date, and was this on the Intensive Care Unit and have you been discharged?
**** If yes, on what date(s) were you exposed to COVID-19?

**Supplementary material C**

Interim analysis for the elderly trial

**References:**

1. WHO. *Coronavirus disease 2019 ( COVID-19 ): Situation Report – 23*. *Covid-19 Situatioanal Reports* (2020).

2. Huang, C. *et al.* Clinical features of patients infected with 2019 novel coronavirus in Wuhan, China. *Lancet* (2020) doi:10.1016/S0140-6736(20)30183-5.

3. Guan, W.-J. *et al.* Clinical Characteristics of Coronavirus Disease 2019 in China. *N. Engl. J. Med.* (2020) doi:10.1056/NEJMoa2002032.

4. Zhang, J. *et al.* Therapeutic and triage strategies for 2019 novel coronavirus disease in fever clinics. *Lancet Respir. Med.* (2020) doi:10.1016/s2213-2600(20)30071-0.

5. Sanders, J. M., Monogue, M. L., Jodlowski, T. Z. & Cutrell, J. B. Pharmacologic Treatments for Coronavirus Disease 2019 (COVID-19): A Review. *Jama* **2019**, (2020).

6. Benn, C. S., Netea, M. G., Selin, L. K. & Aaby, P. A Small Jab - A Big Effect: Nonspecific Immunomodulation By Vaccines. *Trends in Immunology* (2013) doi:10.1016/j.it.2013.04.004.

7. Netea, M. G. *et al.* Trained immunity: A program of innate immune memory in health and disease. *Science* (2016) doi:10.1126/science.aaf1098.

8. Spencer, J. C., Ganguly, R. & Waldman, R. H. Nonspecific protection of mice against influenza virus infection by local or systemic immunization with bacille Calmette-Guerin. *J. Infect. Dis.* (1977) doi:10.1093/infdis/136.2.171.

9. Arts, R. J. W. *et al.* BCG Vaccination Protects against Experimental Viral Infection in Humans through the Induction of Cytokines Associated with Trained Immunity. *Cell Host Microbe* (2018) doi:10.1016/j.chom.2017.12.010.

10. Biering-Sørensen, S. *et al.* Small randomized trial among low-birth-weight children receiving bacillus Calmette-Guéerin vaccination at first health center contact. *Pediatr. Infect. Dis. J.* (2012) doi:10.1097/INF.0b013e3182458289.

11. Kristensen, I., Aaby, P. & Jensen, H. Routine vaccinations and child survival: Follow up study in Guinea-Bissau, West Africa. *Br. Med. J.* (2000) doi:10.1136/bmj.321.7274.1435.

12. Aaby, P. *et al.* Randomized trial of BCG vaccination at birth to low-birth-weight children: Beneficial nonspecific effects in the neonatal period? *J. Infect. Dis.* (2011) doi:10.1093/infdis/jir240.

13. Strategic, Experts, A. G. of & Immunization, O. *Weekly Epidemiological Record (WER)*. (2014).

14. Biering-Sørensen, S. *et al.* Early BCG-Denmark and Neonatal Mortality Among Infants Weighing. *Clin. Infect. Dis. An Off. Publ. Infect. Dis. Soc. Am.* (2017) doi:10.1093/CID/CIX525.

15. Wardhana, Datau, E. A., Sultana, A., Mandang, V. V. & Jim, E. The efficacy of Bacillus Calmette-Guerin vaccinations for the prevention of acute upper respiratory tract infection in the elderly. *Acta Med. Indones.* (2011).

16. World Health Organization. Recommendations to assure the quality , safety and efficacy of BCG vaccines. *Propos. Replace. TRS* (2011).

17. Hatherill, M. *et al.* Safety and reactogenicity of BCG revaccination with isoniazid pretreatment in TST positive adults. *Vaccine* (2014) doi:10.1016/j.vaccine.2014.04.084.

18. Nemes, E. *et al.* Prevention of M. Tuberculosis infection with H4:IC31 vaccine or BCG revaccination. *N. Engl. J. Med.* (2018) doi:10.1056/NEJMoa1714021.

19. ResearchOnline. https://www.researchonline.info/nl-nl/.

20. Castor. https://helpdesk.castoredc.com/article/172-how-do-i-cite-castor-edc-in-my-paper.

21. ResearchFollowApp. https://www.researchfollowapp.com/.

22. Bürkner, P. C. brms: An R package for Bayesian multilevel models using Stan. *J. Stat. Softw.* (2017) doi:10.18637/jss.v080.i01.

23. Fine, J. P. & Gray, R. J. A Proportional Hazards Model for the Subdistribution of a Competing Risk. *J. Am. Stat. Assoc.* (1999) doi:10.1080/01621459.1999.10474144.

24. WHO. Bacille Calmette-Guérin ( BCG ) vaccination and COVID-19. 5–6 (2020) doi:10.3389/fimmu.2018.00869.3.
